# DDB2 Genetic Risk Factor for Ocular Squamous Cell Carcinoma Identified in Three Additional Horse Breeds

**DOI:** 10.3390/genes11121460

**Published:** 2020-12-05

**Authors:** Margo Crausaz, Thomas Launois, Kathryn Smith-Fleming, Annette M. McCoy, Kelly E. Knickelbein, Rebecca R. Bellone

**Affiliations:** 1 Veterinary Genetics Laboratory, School of Veterinary Medicine, University of California-Davis, Davis, CA 95616, USA; mcrausaz@ucdavis.edu; 2 Bailly Vétérinaires Clinique du Lys, 77190 Dammarie-les-Lys, France; thomas.launois@orange.fr; 3 Department of Veterinary Clinical Medicine, University of Illinois, Urbana, IL 61802, USA; kmf5@illinois.edu (K.S.-F.); mccoya@illinois.edu (A.M.M.); 4 Veterinary Medical Teaching Hospital, University of California-Davis, Davis, CA 95616, USA; kknickelbein@ucdavis.edu; 5 Department of Population Health and Reproduction, School of Veterinary Medicine, University of California-Davis, Davis, CA 95616, USA

**Keywords:** horse, genetics, *damage-specific DNA binding protein 2* (*DDB2*), cancer, ocular

## Abstract

Squamous cell carcinoma (SCC) is the most common cancer affecting the equine eye. A missense variant within the gene damage-specific DNA binding protein 2 *(DDB2* c.1013*C>T*, p.Thr338Met) was previously identified as a causal recessive genetic risk factor for the development of ocular SCC within Haflingers, Belgian Draft horses, and Rocky Mountain Horses, but not in the Appaloosa or Arabian breeds. This study aimed to evaluate three cases of ocular SCC in additional breeds and determine if DNA testing for the DDB2 variant in warmblood horses and Connemara ponies is warranted. Histopathology confirmed ocular SCC in all three cases and DNA testing confirmed each horse was homozygous for the DDB2 risk factor. The DDB2 risk allele frequency was estimated to be 0.0043 for Holsteiners (*N* = 115), 0.014 for Belgian Warmbloods (*N* = 71), and 0.22 for Connemara Ponies (*N* = 86). Taken together these data support using DNA testing for DDB2 in Connemara Ponies to assist in mate selection and clinical management. Given the low observed allele frequencies in both the Holsteiner and Belgian Warmblood breeds and that the case under investigation was a warmblood cross-bred, evaluating additional SCC affected warmbloods is warranted to fully determine the importance of DDB2 genotyping as a risk factor in warmblood breeds.

## 1. Introduction

Squamous cell carcinoma (SCC) is the most common cancer impacting the equine eye and follows equine sarcoids as the second most common equine cancer overall [1,2,3]. These tumors primarily arise from the limbus, nictitating membrane, and upper or lower eyelids and prognosis varies with location, progression, and treatment. If left untreated, ocular SCC can lead to visual impairment, damage to ocular and central nervous system structures, and potentially metastasis [4,5,6,7]. Several risk factors have been previously associated with the development of SCC, notably exposure to UV radiation, producing DNA damage and disruption of the p53 pathway. p53 is a well-known tumor-suppressor protein that as a transcription factor is involved in the regulation of cell cycle genes, including those involved in DNA repair [3,4,8,9]. Recently, a major genetic risk factor was identified and connected increased risk related to UV damage in certain horse breeds [4]. Specifically, investigation of the genetic risk for ocular SCC in Haflingers identified a recessive allele in damage-specific DNA binding protein 2 (*DDB2* c.1013*C>T*, p.Thr338Met) [4,10]. The missense mutation (p.Thr338Met) in a conserved β-hairpin loop was hypothesized to impair binding of this protein to UV-damaged DNA. Data from purified recombinant human DDB1-DDB2 complexes containing p.Thr338Met supports this hypothesis as this recombinant protein did not bind DNA as evidenced through electromobility shift assays [11].

DDB2 normally recognizes and binds to cyclobutene pyrimidine dimers (CPD) initiated by UV radiation. Once bound, DDB2 recruits a protein complex to repair the UV damage [4,12]. However, when p.Thr338Met is present, DDB2′s protein conformation is predicted to be altered, thus preventing it from binding to DNA and recognizing UV-induced photolesions [4,11,13,14,15]. Since this mutated DDB2 is unable to bind to DNA, the necessary protein complex for repair would not be recruited, and accumulation of unrepaired UV damage, in horses homozygous for the risk variant, likely predisposes them to the development of ocular SCC.

In investigating the Haflinger breed, the missense variant explained 76% of the limbal and nictitating SCC case cohorts [4,15]. Subsequent work identified the same risk factor in the Belgian Draft horse breed [16]. A case report of a Rocky Mountain Horse supported the DDB2 variant as an ocular SCC risk factor in this breed as well, as the horse afflicted with limbal SCC was homozygous for the risk allele [17], and the allele frequency in Rocky Mountain Horses was similar to that reported for the Haflinger and Belgian Draft horse breeds (0.20 compared to 0.25 and 0.21, respectively) [4,16,17].

The DDB2 risk variant was also identified at a low frequency in the Percheron and Appaloosa breeds (0.07 and 0.02, respectively) [4,18]. Subsequent studies using 46 SCC-affected Appaloosas did not support genotyping for DDB2 in the Appaloosa as none of these cases were homozygous for this risk variant. However, additional work is needed to evaluate if testing for the DDB2 risk allele is warranted in Percherons, as only two horses were identified to have SCC in that study and none were homozygous for the risk allele [18]. Retrospective studies have identified warmblood and pony breeds also as overrepresented for ocular SCC [4,19,20,21,22,23], however, the role of the DDB2 variant in these breeds has not been determined [4,15,16,17,18].

The correlation between pigmentation and the DDB2 genotype has yet to be investigated, however several studies suggest that horses with chestnut coat color and grey depigmentation phenotypes are overrepresented in the occurrence of ocular SCC [4,17,23,24,25]. While hypotheses have been developed to determine how pigmentation plays a role in the development of ocular SCC, further molecular studies are needed to investigate the specific mutations that are leading to the occurrence of SCC in horses [4].

The aim of this study is to report the occurrence of the *DDB2* c.1013*C>T* variant in additional cases and to identify the allele frequency of this variant in larger sample cohorts from the Holsteiner, Belgian Warmblood, and Connemara Pony breeds. These data will help to establish if testing for the risk allele could be utilized for marker-assisted selection and if utilization of genetic testing for this variant by clinicians in management of the disease is warranted in these additional breeds.

## 2. Materials and Methods

### 2.1. Ophthalmic Examination

Three privately owned horses were presented to the Bailly Vétérinaires Clinique de Lys (*N* = 2) and the University of Illinois Veterinary Teaching Hospital (*N* = 1) for evaluation of suspected ocular SCC. Each horse underwent a complete physical examination, and neuro-ophthalmic and ophthalmic examinations, including evaluation of menace response, dazzle reflex, and palpebral reflex. Additionally, eyes were evaluated via ophthalmoscopy and slit lamp biomicroscopy. All ocular tissues were examined for abnormalities, and anomalies were noted. Suspect masses were biopsied and sent for histopathology.

### 2.2. Evaluation of Retrospective Data

The medical records at Bailly Vétérinaires Clinique du Lys (2014–2020) and the University of Illinois Veterinary Teaching Hospital (2015–2020) were searched for horses diagnosed with ocular SCC. Signalment data of horses affected with ocular SCC was collected to determine the breed and sex distribution and frequency of affected cases presenting during this time period. Breed distribution for ocular SCC was also compared to ophthalmology caseload for the same period.

### 2.3. Genetic Testing

For each horse, blood was collected and stored in a vial containing EDTA and approximately 50 hairs were pulled from the mane. Genomic DNA was isolated from whole blood using a DNA extraction kit (Puregene, Qiagen Inc., Valencia, CA, USA) following the manufacturer’s protocol. Following extraction, genotyping for the *DDB2* c.1013 *C>T* risk variant was performed through the University of California, Davis Veterinary Genetics Laboratory (VGL) services [26]. This assay was established based on the initial identification of the variant [4]. Further, positive controls for each genotype and a negative control (no DNA) were performed with each assay. Genotypes were subsequently validated with DNA isolated from the hair follicles. Hair follicles were processed using the same DNA extraction kit with a modified protocol [4]. Genotyping for known variants affecting coat color was also performed through the commercially available assay at the VGL.

In order to determine the allele frequency of *DDB2* c.1013*C>T* in the Connemara pony, Holsteiner, and Belgian warmblood breeds, a random subset of unrelated samples (one degree of separation) obtained from banked DNA at the Bellone Laboratory was utilized (*N* = 86 Connemara Ponies, *N* = 115 Holsteiners, and *N* = 71 Belgian Warmbloods). These horses were not phenotyped for ocular SCC. Genotyping for the DDB2 variant was performed using the commercially available assay at the VGL. Allele and carrier frequencies were calculated for each breed. This study was approved by the University of California, Davis IACUC committee under the protocol number 20146.

## 3. Results

### 3.1. Case Presentations

#### 3.1.1. Case 1

**Figure 1:**
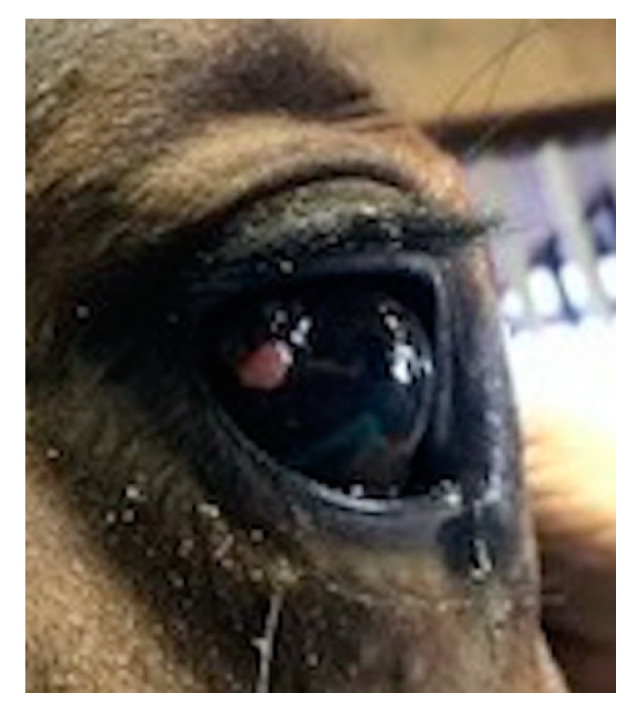
Clinical photograph of right eye of a Holsteiner–Belgian Warmblood cross (Case 1) showing a mass on the temporal bulbar conjunctiva.

A ten-year-old Holsteiner-Belgian Warmblood cross mare was presented to the University of Illinois Veterinary Teaching Hospital with a conjunctival mass on her right eye (OD; Figure 1). Prior to referral, no diagnostics or treatment were performed. Upon ophthalmic examination, an 8 mm by 6 mm raised, partially pigmented mass was found on the bulbar conjunctiva adjacent to the lateral limbus, with no evidence of corneal involvement. The other adnexal structures were normal. No masses were detected on the left eye (OS). The OD mass was excised and submitted for histopathological evaluation, which confirmed conjunctival squamous cell carcinoma in situ.

#### 3.1.2. Case 2

**Figure 2:**
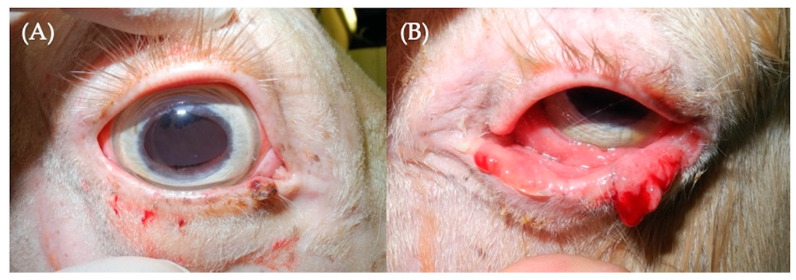
Clinical photographs of the right (**A**) and left (**B**) eyes of a Connemara Pony (Case 2). Note the ulcerated mass on the nasal inferior eyelid OD (**A**) and the extensive masses affecting the lower eyelid OS (**B**).

A seven-year-old Connemara Pony gelding was presented to the Bailly Vétérinaires Clinique du Lys for evaluation of masses affecting both eyes (OU). It was noted that the masses began to grow 8 months prior to presentation. Examination of the right eye revealed a pink mass on the temporal angle of the conjunctiva and an 8 mm diameter, roughly spherical, ulcerated mass on the nasal lower eyelid (Figure 2A). On examination of the left eye, masses were found on the lower eyelid and the temporal cornea and limbus (Figure 2B). The OD masses were excised while an orbital exenteration was performed OS due to the invasive nature of the masses. Histopathology determined the masses to contain moderately differentiated epithelial neoplasia with subsequent squamous differentiation and moderate to marked anisocytosis and anisokaryosis, confirming ocular SCC.

#### 3.1.3. Case 3

**Figure 3:**
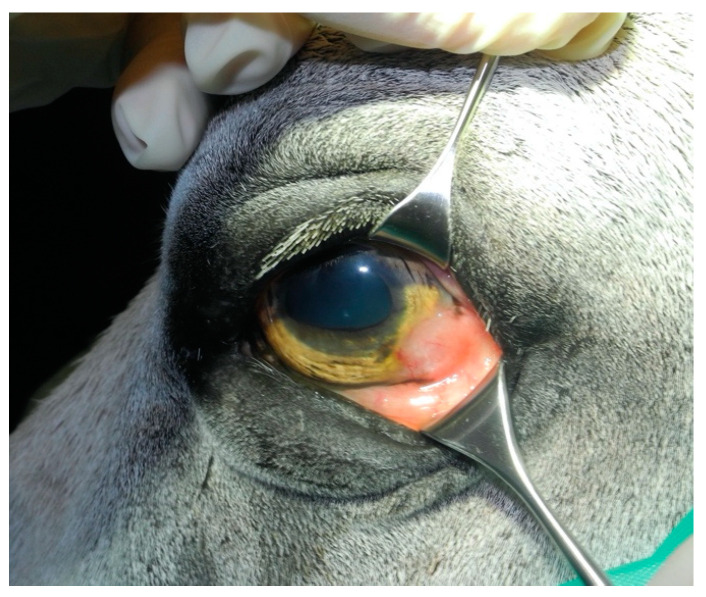
Clinical photograph of a Connemara Pony (Case 3) depicting the pink, raised mass on the left temporal limbus and cornea.

A seventeen-year-old Connemara Pony gelding was presented to the Bailly Vétérinaires Clinique du Lys for evaluation of a mass affecting the left eye. Ophthalmic examination revealed a pink, raised mass affecting the temporal limbus and cornea OS (Figure 3). The mass was excised completely and sent for histopathological examination. Histopathology found the mass to be composed of atypical keratinized epithelial cells that were not uniform (differed in shape from round to polygonal), and were arranged in clusters or individually. Criteria for malignancy were considered to be obvious and included a high nucleocytoplasmic ratio, large nuclei with granular chromatin, prominent and visible nucleoli, moderate anisocytosis, and anisokaryosis. The pathologist’s conclusion was epithelial carcinoma (SCC).

### 3.2. Retrospective Signalment Data

Investigating six years of records from the Bailly Vétérinaires Clinique du Lys in France found that approximately 17% of SCC cases were Connemara Ponies and another 17% were Haflingers. Connemaras and Haflingers made up 0.74% and 1.49% of the overall ophthalmology caseload during this time frame. The warmblood breed Selle Français was also among the top three breeds represented in these data (17%), but was also the most common breed (56%) when comparing breed representation for all ophthalmology cases (Table 1). We also investigated retrospective data from the USA, at the University of Illinois Veterinary Teaching Hospital, over a similar period and only one Holsteiner (the Holsteiner cross reported here) was presented to the hospital, with the majority of SCC cases being American Paint Horses (36%) followed by American Quarter Horses (18%). These two breeds comprised the highest number of ophthalmology cases during this time frame (15.74% and 26.81%, respectively; Table 1). Additionally, approximately 6% of SCC cases were Haflingers, while comprising only 1.28% of the total ophthalmology caseload. In analyzing these data by clinic (location) or as a combined data set, the sex distribution was identical, 66.7% of the cases were geldings with the remaining cases all being mares. Similarly, in either data set the average age of diagnosis slightly varied by breed, with the overall mean age being 14 years and age of diagnosis ranging from 5 to 28 years (Table 1).

### 3.3. Genetic Testing

#### 3.3.1. DDB2 and Coat Color Genotypes

The genotyping results confirmed homozygosity for the DDB2 risk variant for all three cases (Table 2).

Investigating genotypes for coat color loci routinely tested at the VGL identified variation in the coat colors represented with Case 1 being a bay, Case 2 being a perlino, and Case 3 being a gray (Table 3).

#### 3.3.2. Allele frequency of *DDB2* c.1013*C>T* in Connemara Ponies and Holsteiners

Genotyping for *DDB2* c.1013*C>T* estimated the risk allele frequency to be f(A) = 0.22 in Connemara Ponies. Of the 86 additional individuals, 6 were homozygous for *DDB2* c.1013*C>T* and 25 individuals were heterozygous (Table 4). The sample set was found to have a carrier frequency of 0.29. In Holsteiners, estimated *DDB2* c.1013*C>T* allele frequency was f(A) = 0.0043. Of the 115 individuals evaluated in the random sample set, none were homozygous for *DDB2* c.1013*C>T* and only 1 individual was heterozygous (Table 4). The sample set was found to have a low carrier frequency of 0.0087. In the Belgian Warmblood, the allele frequency was estimated at f(A) = 0.014 with only 2 out of 71 horses being heterozygous and no homozygotes detected.

## 4. Discussion

This report documents three cases of SCC within the Connemara Pony (two cases) and warmblood breeds (one case). All three horses were found to be homozygous for the *DDB2* c.1013 *C>T* variant, a known genetic risk factor for SCC in Haflingers, Belgian Draft horses, and Rocky Mountain Horses, but not previously investigated in pony or warmblood breeds [4,16,17]. Sequencing of DDB2 was performed previously in Haflinger and Belgian Draft horses and while a total of five other missense variants exist in the public domain, only *DDB2* c.1013 *C>T* was predicted to alter protein function (Table 5) [4,16]. The aim of this work was to determine if the known risk variant (*DDB2* c.1013*C>T*) could have led to the development of SCC in the affected Holsteiner cross and the two affected Connemara Ponies and these data support this to be the case.

Other potential risk factors for SCC development have been studied previously, including age, sex, coat color, and equine papilloma virus type 2 infection [18]. While risk for ocular SCC has been highly correlated with the DDB2 genotype, not all cases of ocular SCC were explained by homozygosity for this variant. Genetic heterogeneity is likely involved [18]. In our study, the mean age of diagnosis from two retrospective data sets was found to be 14.11 ± 5.39 years, consistent with what is reported in the literature [10]. In the present study, Case 2 was younger than the reported [10] and our study mean age of diagnosis (even considering one standard deviation), suggesting that potentially other genetic risk factors may be involved. Previous research has implicated pigmentation type, dilution, or lack thereof, as another risk factor, specifically horses that were chestnut (pheomelanin production only), dilute shades of chestnut, or gray, and horses lacking periocular pigment were overrepresented in retrospective case studies [4,23,24,25]. Amount of UV exposure has also been documented as a risk factor [8]. Therefore, it is possible that there is an increased risk associated with high amounts of UV exposure, lack of functional DDB2, and the type and/or amount of periocular pigment. The interaction of these risk variables remains to be evaluated. Overrepresentation of horses lacking periocular pigment is supported in the retrospective data from the University of Illinois, with Paint Horses comprising 36.36% of SCC cases while only representing 15.74% of the total ophthalmology caseload at this institution over the study time frame. This breed is known for its white spotting patterns, which often lack pigment around the eyes. Investigating if there is a particular white spotting allele contributing to ocular cancer risk remains to be evaluated. Additionally, Case 2 genotyped as a bay horse with two copies of the cream color dilution mutation, demonstrating a perlino coat phenotype. The mutation responsible for the cream pigment dilution is in the gene membrane associated transport protein (*MATP*) and is thought to disrupt pigmentation by impairing normal trafficking of pigment granules within the developing melanocyte [28]. Horses homozygous for the cream dilution typically have a very dilute coat, lack pigment in their skin, and have blue irises, as presented in Case 2 [28]. Thus, a combination of reduced photoprotective pigment and loss of functional DDB2 could have contributed to the younger age and bilateral nature of SCC development, and aggressive clinical course that necessitated exenteration. Investigating additional horses with the *MATP* variant, in combination with their extent of UV exposure, would help to better understand the relationship between DDB2 and variants that dilute pigment, and how this impacts SCC risk and disease progression. Case 3, who was 17 when diagnosed, was older than the reported mean age, but also displayed a gray coat color, which is the progressive loss of pigment caused by a 4.6 kilobase duplication in an intron of the syntaxin 17 (*STX17*) gene [29]. Previous research found gray to be among the most frequent coat color of SCC-affected cases [25], yet how progressive loss of pigment in combination with the DDB2 loss of function mutation impacts the risk for SCC remains to be evaluated. In our prior studies, all horses investigated were chestnut, so further evaluation of risk associated with multiple mutations in photoprotective pigment genes in combination with DDB2 genotyping and evaluation of extent of UV exposure should be explored.

Previous research has also implicated hormones as a risk factor for ocular SCC with geldings being overrepresented compared to stallions and mares in retrospective studies [3,4,18,23,24,30]. It has been proposed that lack of circulating androgens and estrogens in castrated males predispose geldings to ocular SCC [8,18]. Our retrospective data provides further support that 66.7% of ocular SCC cases were geldings. In a previous study, involving Haflingers, there was no statistical difference in the number of males and females with regard for SCC status however, significantly more geldings were affected than stallions compared to the control sample set [4,18]. The impact of the DDB2 genotype and castration status on ocular SCC risk has not been investigated. In this study, both Connemara Pony cases were homozygous for the DDB2 variant and were geldings. A larger sample set of affected Connemara Ponies along with a well phenotype cohort of controls genotyped for DDB2 is needed to evaluate any potential increased risk associated with being a gelding homozygous for the DDB2 risk variant.

While the allele frequency for the *DDB2* c.1013*C>T* variant for Holsteiner and Belgian Warmblood horses was determined to be relatively low (f(A) = 0.0043 and 0.014 respectively), these sample sets of horses were not available to be phenotyped for ocular SCC. While the likelihood of a purebred Holsteiner or Belgian Warmblood being *T/T* is low, as estimated by the allele frequency of *DDB2* c.1013 *C>T,* the potential of a Holsteiner being a carrier was approximately 9 horses in a 1000 and was greater (approximately 3 in a 100) for the Belgian Warmblood. Due to the presence of the risk allele in Holsteiner and Belgian Warmblood breeds, this variant may be an important genetic risk factor for ocular SCC in multiple warmblood breeds. As the most common breed examined for ocular issues in France, retrospective data also support the evaluation of the Selle Français for genetic risk factors for ocular SCC. Clinical phenotyping of warmblood horses from diverse breeds, including Selle Français, along with DDB2 DNA testing, should be performed to further evaluate if loss of function mutations in DDB2 and UV-induced damage also explain increased risk for ocular SCC in warmblood horse breeds.

The allele frequency for the *DDB2* c.1013*C>T* variant within the Connemara Pony was calculated to be 0.22. This is similar to that of the Haflingers, Belgian Drafts, and Rocky Mountain Horse (0.25, 0.21, and 0.20, respectively) [4,16,17]. Further, retrospective data presented here demonstrate a similar prevalence of SCC in Connemara Ponies and Haflingers (17% of cases in each breed) from the same geographic location, despite the low representation of these two breeds when considering all the ophthalmology cases within that data set. Estimates of carrier frequencies suggest that 3 in 10 Connemara Ponies are carriers for the DDB2 risk factor. Recent functional evidence supports that DDB2 p.Thr338Met is a loss of function mutation impairing binding of the protein to DNA [11]. While only two SCC-affected Connemara Ponies were evaluated in this study, given the previous associations, evidence of loss of function, prevalence of SCC in Connemara Ponies from retrospective data, and a moderate allele frequency within this breed, this variant is likely an important risk factor in the Connemara Pony breed. Therefore, genetic testing for this variant is recommended as it is for the Haflinger, Belgian Draft Horse, and Rocky Mountain Horse breeds.

The DDB2 risk variant has now been identified and connected with ocular SCC in six distantly related horse breeds. Clinically evaluating horses for ocular SCC by examination and histopathology from breeds commonly presenting with ocular SCC, including the Selle Français and the American Paint Horse identified from the retrospective data presented in this report, in addition to genotyping the DDB2 variant in a diverse breed sample set phenotyped for ocular SCC, would assist in a better understanding of the breed distribution and connection to ocular SCC across equine breeds. Establishing breed-specific recommendations with regard to genetic testing would allow for the best use of this genetic test for mate selection in order to decrease the incidence of disease and identify which horses are at highest risk of developing ocular SCC so they can be monitored for early detection, increasing the likelihood of a more favorable outcome.

## Figures and Tables

**Table 1:** Retrospective breed distribution of ocular squamous cell carcinoma (SCC) cases at two veterinary clinics.

Veterinary Clinic	Breed	Number of Cases (Percent of Cases)	Percent of Total Ophthalmology Caseload	Number of Geldings	Number of Mares	Average Age of Diagnosis
**Bailly Vétérinaires Clinique du Lys**	Haflinger	4 (16.7%)	1.49%	4	0	10 ± 4.97
	Connemara	4 (16.7%)	0.74%	4	0	15.75 ± 6.08
	Selle Français	4 (16.7%)	56%	2	2	7.50 ± 2.89
	Trotteur Français	2 (8.3%)	2.23%	2	0	12 ± 2.83
	Draft horse	1 (4.2%)	0.2%	0	1	15
	Paint Horse	1 (4.2%)	0.2%	0	1	11
	Noriker Draft Horse	1 (4.2%)	0.2%	0	1	14
	Anglo Arabian	1 (4.2%)	0.2%	1	0	9
	Appaloosa	1 (4.2%)	2.98%	0	1	17
	Arabian–Boulonnais cross	1 (4.2%)	0.2%	1	0	12
	Unknown Breed	1 (4.2%)	0.2%	0	1	16
	Trait Breton	1 (4.2%)	0.2%	0	1	N/A
	Irish Cob	1 (4.2%)	0.2%	1	0	14
	Welsh	1 (4.2%)	0.2%	1	0	13
**Totals**		**24**		**16 (66.7%)**	**8 (33.3%)**	**12.09 ^1^** **± 4.47**
**University of Illinois Veterinary Teaching Hospital**	Paint Horse	12 (36.36%)	15.74%	11	1	13.67 ± 4.01
	Quarter Horse	6 (18.18%)	26.81%	3	3	17.83 ± 7.22
	Appaloosa	4 (12.12%)	5.96%	3	1	21 ± 5.35
	Arabian	3 (9.09%)	6.38%	2	1	16 ± 4.58
	Haflinger	2 (6.06%)	1.28%	0	2	13 ± 1.41
	Mixed Breed Horse	2 (6.06%)	2.98%	1	1	16 ± 7.07
	Holsteiner–Belgian Warmblood cross	1 (3.03%)	0.43%	0	1	10
	Clydesdale	1 (3.03%)	0.85%	1	0	23
	Tennessee Walker	1 (3.03%)	4.68%	1	0	9
	Thoroughbred	1 (3.03%)	7.23%	0	1	9
**Totals**		**33**		**22 (66.7%)**	**11 (33.3%)**	**15.52** **± 5.58**
**Cumulative Totals**		**57**		**38 (66.7%)**	**19 (33.3%)**	**14.11 ^1^** **± 5.39**

^1^ Excluding Trait Breton from the total average age of diagnosis, as age was not available.

**Table 2:** *DDB2* c.1013 *C>T* genotype results determined for the three SCC cases under investigation.

Case Number	Breed	Age (Years)	Ocular SCC	*DDB2* c.1013*C>T* Genotype
Case 1	Holsteiner–Belgian Warmblood cross	10	Conjunctival in situ OD	*T/T*
Case 2	Connemara Pony	7	Eyelid OD, Limbal and Eyelid OS	*T/T*
Case 3	Connemara Pony	17	Limbal OS	*T/T*

**Table 3:** Coat color genotype results determined for the three ocular SCC cases.

Case Number	Red Factor	Agouti	Cream	Pearl	Silver	Dun	Champagne	White Spotting Pattern Tests ^1^	Gray	Coat Color
Case 1	E/E	A/A	N/N	N/N	N/N	nd2/nd2	N/N	N/N	N/N	Bay
Case 2	E/E	A/a	Cr/Cr	N/N	N/N	nd1/nd2	N/N	N/N	N/N	Perlino
Case 3	E/E	A/A	N/Cr	N/N	N/N	nd2/nd2	N/N	N/PATN1 ^2^	G/G	Gray

^1^ White Spotting Pattern Tests include: Lethal White Overo, Sabino 1, Dominant White (W5, W10, W20), Splashed White (SW1, SW3), Splashed White (SW2, SW4), Tobiano, Leopard, Pattern-1). ^2^ N/N for every other pattern test except Pattern-1, where one copy of PATN1 was detected.

**Table 4:** *DDB2* c.1013*C>T* allelic frequencies and genotypic frequencies in the Connemara pony and Holsteiner breeds.

Breed	Sample Size	Genotypic Frequency *T/T*	Genotypic Frequency *C/T*	Genotypic Frequency *C/C*	*DDB2* c.1013*C>T* Allele Frequency	*T/T*	*C/T*	*C/C*
Connemara Pony	86	0.070 ^1^	0.29 ^1^	0.64 ^1^	0.22 ^1^	6	25	55
Holsteiner	115	0	0.0087 ^1^	0.99 ^1^	0.0043 ^1^	0	1	114
Belgian Warmblood	71	0	0.028 ^1^	0.97 ^1^	0.014 ^1^	0	2	69

^1^ Data rounded to 2 significant figures.

**Table 5:** Missense variants identified in damage-specific DNA binding protein 2 (*DDB2*).

Variant Id	Position	Ensembl Transcript ID	SNP ID	SIFT Score (SIFT Class)	Reference
rs68921635	12:11728317	ENSECAT00000058569.1	*DDB2* c.1230G>C p.Leu410Phe	0.53 (tolerated—low confidence)	[4,16]
rs1136080479	12:11707134	ENSECAT00000009017.2	*DDB2* c.379C>G p.Leu127Val	0.61 (tolerated)	[27]
rs1147672039	12:11726162	ENSECAT00000009017.2	*DDB2* c.835T>G p.Ser279Ala	0.52 (tolerated)	[27]
rs1151711657	12:11707119	ENSECAT00000009017.2	*DDB2* c.364G>A p.Ala122Thr	1 (tolerated)	[27]
rs782834602	12:11726610	ENSECAT00000009017.2	*DDB2* c.956A>G p.Gln319Arg	0.31 (tolerated)	[4,16]
rs1139682898	12:11726667	ENSECAT00000009017.2	*DDB2* c.1013C>T p.Thr338Met	0 (deleterious)	[4,16]
